# Acute Caffeine Ingestion did not Enhance Punch Performance in Professional Mixed-Martial Arts Athletes

**DOI:** 10.3390/nu11061422

**Published:** 2019-06-25

**Authors:** Arthur Persio de Azevedo, Mauro Antônio Guerra, Leonardo Carvalho Caldas, Lucas Guimarães-Ferreira

**Affiliations:** 1 Postgraduate Program in Nutrition and Health, Centre of Health Sciences, Federal University of Espirito Santo, Vitória-ES 29075-910, Brazil; persio28@hotmail.com; 2 Muscle Physiology and Human Performance Research Group, Center of Physical Education and Sports, Federal University of Espirito Santo, Vitória-ES 29075-910, Brazil; guerrajr2@yahoo.com.br (M.A.G.J.); leocaldas03@gmail.com (L.C.C.); 3 Postgraduate Program in Physical Education, Center of Physical Education and Sports, Federal University of Espirito Santo, Vitória-ES 29075-910, Brazil

**Keywords:** caffeine, performance, combat sports, mixed martial arts

## Abstract

Mixed martial arts (MMA) is a combat sport where competitors utilize strikes (punches, kicks, knees, and elbows) and submission techniques to defeat opponents in a cage or ring. The aim of this study was to investigate the effect of acute caffeine ingestion on punching performance by professional MMA athletes. The study used a double-blind, counterbalanced, crossover design. Eleven professional MMA competitors (27.6 ± 4.3 years and 83.5 ± 7.8 kg of body weight) ingested a dose of caffeine (5 mg·kg^−1^) or placebo 60 min prior to three sets of punching. Each set consisted of 15 s, at which participants were asked to perform straight punches with maximum strength and frequency with his dominant arm. After each set, a 45 s recovery time was applied. Using a force transducer attached to a cushioned plate, the punch frequency, and mean and maximal punch force was measured. The readiness to invest in both physical (RTIPE) and mental (RTIME) effort was assessed prior to the protocol, and the rating of perceived exertion (RPE) was recorded after. Caffeine ingestion did not result in increased punching frequency, mean and maximum punch force, RTIPE, RTIME, and RPE when compared to the placebo condition. Based on these results, acute caffeine ingestion did not improve punching performance in professional MMA athletes.

## 1. Introduction

Caffeine, or 1,3,7-trimethylxantine, is commonly found in food and medications, and is one of the most consumed supplements worldwide. Due to its both hydrophilic and lipophilic nature, caffeine circulates in the bloodstream and readily crosses phospholipid membranes and the blood-brain barrier, reaching fluid compartments and tissues throughout the body [1]. Studies investigating the effects of caffeine ingestion on sports performance started in the 20th century, but it was in the 1970s when studies on the ergogenic effects of caffeine intensified. It was demonstrated that the ingestion of 330 mg of caffeine 60 min prior to an exercise bout at 80% VO2max resulted in a 19.2% increase in time to exhaustion when compared to the placebo condition [2].

In recent years, investigations were also performed testing the effect of acute caffeine ingestion on high-intensity exercise, where anaerobic pathways for ATP production are predominant, but the number of studies is scarcer and often presents controversial results (reviewed in [3]). For example, Duncan and Oxford [4] investigated the effects of caffeine ingestion (5 mg·kg^−1^) on resistance exercise performance. In the caffeine condition, participants presented an increased capacity to perform repetitions at 60% of the 1 repetition maximum (RM) until concentric failure in the horizontal bench press exercise, when compared to the control group, in addition to lower levels of perception of fatigue and pain. Similarly, Silva et al. [5] demonstrated that caffeine intake (5 mg·kg^−1^) by moderately strength trained individuals resulted in a greater ability to perform repetitions until concentric failure in the horizontal and leg press exercises in consecutive series to 80% of the 1 RM.

On the other hand, some studies have failed to demonstrate the effects of acute caffeine intake on performance in strength training. Hendrix et al. [6] demonstrated that ingestion of a caffeine-containing supplement (400 mg) was not effective in improving performance at the 1 RM in the supine and leg press exercises, or even in the time to exercise exhaustion on the high-intensity cycle ergometer (80% of VO2max). Similarly, Green et al. [7] also reported no difference in the maximal repetition test in the supine and leg press in men and women after the ingestion of 6 mg·kg^−1^ of caffeine when compared to the placebo condition. Such discrepancies may be due to different protocols used (modality, intensity, and duration of the effort), as well as characteristics of the participants (training level and individual responses to caffeine).

Combat sports are characterized by intermittent high-intensity efforts [8,9,10]. Mixed martial arts (MMA) is a combat sport, which combines strikes (punches, kicks, knees, and elbows) and submission techniques. A recent systematic review concluded that acute caffeine ingestion by martial arts practitioners seems to improve strength and power, increasing glycolytic contribution to energy metabolism [10]. Punching performance is important to MMA athletes, as well as for competitors of karate, boxing, and Muay Thai, among other modalities of combat sports. To the best of our knowledge, the effects of caffeine ingestion on punching performance is unknown. To fill this gap, the objective of the current study is to evaluate the effect of acute caffeine ingestion (5 mg·kg^−1^) on punching performance (punching force and frequency) and perceptual responses to a repeated punching protocol in professional MMA athletes.

## 2. Materials and Methods

### 2.1. Participants

Eleven male professional MMA athletes (27.6 ± 4.3 years and 83.5 ± 7.8 kg of body weight) were recruited from two MMA academies. Habitual caffeine consumption was accessed through a questionnaire based on data presented by Maughan [11]. To better control possible individual differences in response to caffeine due do habituation, only participants with a daily caffeine intake of less than 250 mg·d^−1^ were included. The institution’s Human Research Ethics Committee approved the procedures used in this study and all the athletes gave informed consent to take part, in accordance with the 1964 Declaration of Helsinki.

### 2.2. Design and Procedures

For this study, a within-subjects, repeated-measures and double-blinded controlled design was employed. The investigation aimed to examine the effect of acute caffeine ingestion on punch performance (force and frequency). On the first visit, the details on the study were explained to the participants and they executed the testing protocol as familiarization. In two subsequent visits (48 h apart) they performed the testing protocol in two conditions: 60 min after the ingestion of caffeine (5 mg·kg^−1^ diluted in 400 mL of flavored solution) or placebo (400 mL of flavored solution). The solutions were identical in flavor and color. The participants were asked to follow the same diet and exercise practices before each trial but to abstain from caffeine consumption (in drinks and supplements) during all data collection period.

The punching exercise protocol was adapted from Donovan et al. [12] and consisted of three sets of punching on a padded plate attached to a force transducer of 200 kgf (CEFISE©, São Paulo, Brazil). Each set consisted of 15 s, at which participants were asked to perform straight punches with maximum strength and frequency with his dominant arm. After each set, a 45 s recovery time was applied. The number of punches and the force of each punch was recorded, and the maximal force, mean force and frequency (number of punches per set) were calculated. The participants were asked to rate how physically (RTIPE) and mentally (RITME) ready they were to invest effort using visual scales ranging from 0 to 10, with higher scores reflecting a greater readiness to invest effort, as used before [4]. After the protocol, the rating of perceived exertion (RPE) was assessed using a 6–20 scale, with 6 reflecting minimum effort and 20 maximal effort [13].

### 2.3. Statistical Analysis

The maximal and mean punch forces and punch frequency per set were assessed using a 2-way (condition: Placebo vs. caffeine) × 3 (sets) repeated measures analysis of variance (ANOVA). Post hoc analysis using Bonferroni adjustments were performed where any significant interactions and main effects were found. Total punch frequency, RPE, RTIPE, RTIME were analyzed using paired sample *t*-tests for each variable. A *p*-value of 0.05 was used to establish statistical significance. The statistical software GraphPad Prism version 6.0 (GraphPad Software, Inc., San Diego, CA, USA) was used for all analysis.

## 3. Results

**Figure 1:**
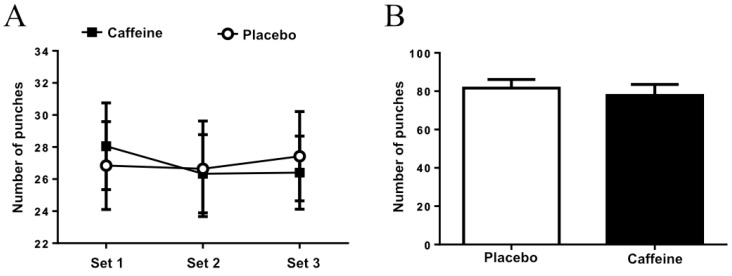
(**A**) Punch frequency at each set. (**B**) Total number of punches executed during three sets.

Caffeine ingestion did not result in a higher punch frequency during the three sets (set main effect: *p* = 0.4562; caffeine main effect: *p* = 0.2038; interaction: *p* = 0.2038; Figure 1A), as well as in the total number of punches executed (*p* = 0.99; Figure 1B) when compared to placebo condition.

**Figure 2:**
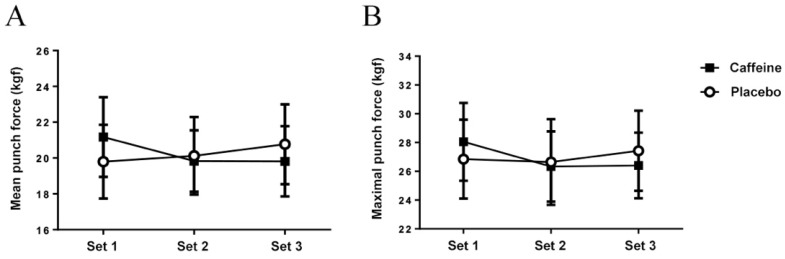
(**A**) Mean punching force. (**B**) Maximum punch force.

Caffeine ingestion did not increase the mean punching force (set main effect: *p* = 0.704; caffeine main effect: 0,953; interaction: *p* = 0.121; Figure 2A) or maximum punching force (set main effect: *p* = 0.453; caffeine main effect: *p*= 0.974; interaction: *p* = 0.367; Figure 2B) during three sets of the repeated punching protocol when compared to placebo condition.

**Figure 3:**
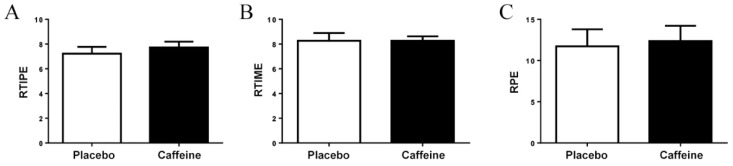
(**A**) Readiness to invest in physical effort; (**B**) Readiness to invest in mental effort; (**C**) Rating of perceived exertion.

The readiness to invest in physical and mental effort (*p* = 0.424 for Figure 3A and *p* = 0.99 for Figure 3B, respectively) was measured before and the RPE (*p* = 0.283, Figure 3C) was accessed after the punching protocol, but no difference was found between caffeine and placebo conditions.

## 4. Discussion

This study examined whether acute caffeine ingestion enhances punching performance and cognitive responses in professional MMA athletes. The results suggest that caffeine supplementation does not exert an ergogenic role in the protocol used herein, since no difference was found between the placebo and caffeine conditions in all evaluated variables. To our knowledge, no study has evaluated the effect of caffeine on punch performance in combat sports athletes. Several studies, since the 1970s, have pointed to an ergogenic effect of caffeine on endurance continuous activities [2,14], although more recent studies also point to a performance improvement effect in high strength and intermittent activities (such as repeated sprints) [15,16]. However, especially regarding high-intensity activities, the results are conflicting, since many studies have failed to demonstrate positive effects of acute caffeine intake on performance.

The use of caffeine as an ergogenic aid for combat sports athletes was investigated. For example, Pereira et al. [17] investigated the effect of caffeine ingestion (6 mg·kg^−1^) by judo athletes using a specific test consisting of performing a throwing technique repeatedly with the highest speed (the Special Judo Fitness Test: SJFT). In that study, the number of throws per set did not differ between the caffeine and placebo conditions. Arazi et al. [18] evaluated 10 female athletes (16.8 ± 1.23 years of age), on maximum strength, muscular endurance (at 60% of the 1 RM), lower limb power and repeated sprints (RAST test) on three occasions after the ingestion of placebo or caffeine in two dosages (2 and 5 mg·kg^−1^). The authors found no positive effects on performance in any of the tests used.

In contrast, Diaz-Lara et al. [19,20] evaluated 14 jiu-jitsu athletes (in each of two studies) after caffeine (3 mg·kg^−1^) or placebo ingestion in several tests: Manual picking, jumping countermovement and various force tests. The authors concluded that previous caffeine ingestion resulted in increased isometric and dynamic muscle strength, strength and power. It is worth mentioning that, although the valences evaluated by the authors are important for jiu-jitsu competitors, the performance in intermittent high-intensity activity was not evaluated, which differs from the present study and that of Pereira et al. [17]. The study by Astley et al. [21] used 18 young judo fighters (16.1 ± 1.4 years) who underwent the SJFT test protocol under placebo and caffeine conditions (4 mg·kg^−1^). In the caffeine condition, the number of throws performed was higher in the caffeine condition when compared to the placebo (29.0 ± 2.6 and 22.1 ± 3.4, respectively), showing a positive effect of caffeine intake on the performance of young judo athletes. Despite the same test protocol being used, the differences of participant characteristics (adults versus young) make it difficult to perform comparisons between the studies of Astley et al. [21] and Pereira et al. [17]. Due to the scarce number of studies evaluating the effect of caffeine ingestion on combat sport performance, as well as the numerous differences between experimental protocols and participant characteristics, it is still difficult to draw conclusions about the ergogenic potential of caffeine for martial arts and combat sports.

In our knowledge, this is the first study to evaluate punch performance in response to caffeine ingestion. Acute caffeine ingestion seems to increase strength and power [22]. It was demonstrated in male and female karate athletes that maximal strength and power (both upper and lower body) were positively correlated with punch acceleration [23]. Moreover, Roschel et al. [24] demonstrated that, compared to defeated athletes, winners presented higher levels of both upper and lower body muscle power. Based on those data, strategies for strength and power development can exert a positive impact on punch performance and, ultimately, increase fight performance in combat sports athletes. Although it was stated that caffeine ingestion increases glycolytic contribution to energy metabolism during combats and improves strength and power [25], we did not find punch performance enhancement after caffeine ingestion. The controversial results between the current study, Pereira et al. [18] and Arazi et al. [18], that failed to find ergogenic effects of acute caffeine ingestion on physical performance in combat sports athletes, in opposition to those that observed increased performance [19,20,21], may be explained by gender and age differences among participants and the tests used.

In addition to RPE, mental readiness and physical readiness for exercise engagement were also used as tools to measure psychological response, as suggested by Tenenbaum et al. [26,27]. Previous studies report that caffeine intake, at the same dose used in the present study, has resulted in increased mental readiness for exertion, without altering physical readiness. For example, Da Silva et al. [4] demonstrated that the ingestion of 5 mg·kg^−1^ of caffeine 60 min before performing a repeated series of strength exercises resulted in an 29% increase in mental readiness when compared to the placebo condition. A striking difference between the study by Silva et al. [4] and the present one concerns the profile of the sample, specifically the training status of the participants. The sample used in the present study was composed of highly trained MMA athletes. It is possible that this explains the absence of a caffeine effect on the mental readiness in the participants, since they already presented a higher mental readiness when compared to the data presented by Da Silva et al. [4] with moderately resistance trained men.

## 5. Conclusions

Acute caffeine ingestion (5 mg·kg^−1^) did not improve punching performance, as measured as punching frequency and force, in professional MMA athletes. The readiness to invest in both physical and mental effort and ratings of perceived exertion were similar in caffeine and placebo conditions. Although previous studies reported improvements in high-intensity intermittent performance after caffeine ingestion, the results presented by the literature are controversial. Differences in the sample, as well as the methodology used, may explain the divergent results. To the best of our knowledge, this study is the first evaluating the effects of caffeine ingestion on repeated punching performance in combat sports athletes.
